# Physicochemical, technofunctional, *in vitro* antioxidant, and *in situ* muscle protein synthesis properties of a sprat (*Sprattus sprattus*) protein hydrolysate

**DOI:** 10.3389/fnut.2023.1197274

**Published:** 2023-06-23

**Authors:** Niloofar Shekoohi, Azza Silotry Naik, Miryam Amigo-Benavent, Pádraigín A. Harnedy-Rothwell, Brian P. Carson, Richard J. FitzGerald

**Affiliations:** ^1^Department of Biological Sciences, University of Limerick, Limerick, Ireland; ^2^Health Research Institute, University of Limerick, Limerick, Ireland; ^3^Department of Physical Education and Sport Sciences, Faculty of Education and Health Sciences, University of Limerick, Limerick, Ireland

**Keywords:** sprat protein hydrolysates, technofunctional, antioxidant, C2C12 cells, muscle growth, muscle protein synthesis

## Abstract

**Introduction:**

Sprat (*Sprattus sprattus*) is an underutilized fish species that may act as an economic and sustainable alternative source of protein due to its good amino acid (AA) profile along with its potential to act as a source of multiple bioactive peptide sequences.

**Method and results:**

This study characterized the physicochemical, technofunctional, and *in vitro* antioxidant properties along with the AA profile and score of a sprat protein enzymatic hydrolysate (SPH). Furthermore, the impact of the SPH on the growth, proliferation, and muscle protein synthesis (MPS) in skeletal muscle (C2C12) myotubes was examined. The SPH displayed good solubility and emulsion stabilization properties containing all essential and non-essential AAs. Limited additional hydrolysis was observed following *in vitro-*simulated gastrointestinal digestion (SGID) of the SPH. The SGID-treated SPH (SPH-SGID) displayed *in vitro* oxygen radical antioxidant capacity (ORAC) activity (549.42 μmol TE/g sample) and the ability to reduce (68%) reactive oxygen species (ROS) production in C2C12 myotubes. Muscle growth and myotube thickness were analyzed using an xCELLigence™ platform in C2C12 myotubes treated with 1 mg protein equivalent.mL^−1^ of SPH-SGID for 4 h. Anabolic signaling (phosphorylation of mTOR, rpS6, and 4E-BP1) and MPS (measured by puromycin incorporation) were assessed using immunoblotting. SPH-SGID significantly increased myotube thickness (*p* < 0.0001) compared to the negative control (cells grown in AA and serum-free medium). MPS was also significantly higher after incubation with SPH-SGID compared with the negative control (*p* < 0.05).

**Conclusions:**

These preliminary *in situ* results indicate that SPH may have the ability to promote muscle enhancement. *In vivo* human studies are required to verify these findings.

## Introduction

According to the Food and Agriculture Organization (FAO), there is an increasing demand for high-quality protein due to the growth of the global population (1). Fish and shellfish are excellent sources of high-quality protein, e.g., a 100 g cooked serving of most types of fish provides ~18–20 g of protein (2). Global production of aquatic animals was estimated at 178 million tons in 2020. Of the total production, 112 million tons was harvested in marine waters (3). The extraction of food ingredients from discarded fish and fish processing co-products by using enzyme technology or protein recovery has the potential to add value by enhancing and upgrading marine proteins (4). This approach utilizes marine by-products and secondary raw materials, and in addition, it increases the responsible use of low-value/underutilized fish species which are a rich source of high-quality protein (5–7). Furthermore, bioactive protein hydrolysates/peptides derived from low-value/underutilized protein sources have potential applications as high-value functional food ingredients (8). Therefore, the generation of fish protein/peptide ingredients has the potential to unlock new added value opportunities for the fish processing sector promoting its economic and environmental efficiency (9, 10).

Sprat (*Sprattus sprattus* L.) is a small oily fish which is a good source of vitamins, minerals, and proteins (11). Sprat is extensively utilized for human consumption in Eastern European and Scandinavian countries; however, it is currently generally underutilized for human use in other countries (11). In 2022, the volume of sprat produced by the Irish seafood sector was 7,200 tons (12). The production of food protein ingredients derived from sprat represents a good opportunity to add value to this resource and to increase the overall profitability and sustainability of the fisheries sector. Enzymatic hydrolysis of anchovy sprat (*Clupeonellaengrauliformis*) and sprat (*Sprattus sprattus*) proteins has been shown to lead to an improvement in their nutritional (protein content and amino acid (AA) composition) and in their *in vitro* antioxidant properties (13–15). It has been reported that humans can benefit from the ingestion of fish-derived protein hydrolysates (FPHs) to support healthy aging, metabolic health, and skeletal muscle metabolism (16). Because of their high-quality protein content and associated peptides, FPHs may be useful as alternative protein sources (instead of dairy proteins such as whey protein) and as functional foods with the ability to prevent muscle atrophy leading to improvements in muscle mass, strength, and function, particularly in the aging population (16). The consumption of 150–170 g of fish twice per week for a 10-week period has been reported to improve muscle mass and function and may potentially decrease sarcopenia progression in middle-aged and older adults (17). Cordeiro et al. (18) reported that the consumption of 0.25 g/kg body mass of FPH (Nile tilapia-derived) induced immediate and robust post-exercise total aminoacidemia similar to a whey protein hydrolysate, which has been demonstrated to enhance MPS in humans (18).

In addition to the biofunctional value achievable during enzymatic hydrolysis of sprat protein, it is important to explore the technofunctional properties of the hydrolysate to understand its potential applications in the food industry. Minimum or no information seems to exist in relation to the technofunctional properties of SPHs. FPHs generated from other fish species, such as Ribbonfish (*Lepturacanthus savala*) and Yellow stripe trevally (*Selaroides leptolepis*), are reported to have enhanced solubility and oil-holding capacity in a degree of hydrolysis (DH)-dependent manner (19). However, while the solubility increased with increasing DH, higher DH values led to a decrease in the emulsion and foam properties of a yellow stripe trevally hydrolysate (20).

To the best of our knowledge, no information appears to exist in relation to sprat protein hydrolysates (SPH), i.e., their bioactivity and technofunctional properties. Therefore, the aim of this study was to assess the physicochemical, technofunctional, and *in vitro* and *in situ* antioxidant properties of an SPH.

Moreover, the MPS-stimulating ability of the SPH in murine C2C12 cells was examined, with the view to finding alternative protein sources for enhancement of muscle health. Generally, the knowledge of these properties is relevant to the utilization of the hydrolysate in final food products targeted at muscle health maintenance.

## Materials and methods

###  Chemicals and reagents

Kjeldahl catalyst tablets (free of Hg and Se), boric acid, sulfuric acid (≥ 98%), pepsin (P6887, ≥3,200 units/mg protein), pancreatin (P7545, 4 × USP specification), 6-hydroxy-2,5,7,8-tetramethylchromane-2-carboxylic acid (Trolox), 2′, 7′-dichlorodihydrofluorescein-diacetate (DCFH-DA) and 2,2′-azobis-(2-methylpropianomidine) dihydrochloride (AAPH), Dulbecco's Modified Eagles Medium (DMEM), penicillin/streptomycin, fetal bovine serum (FBS), horse serum, Hank' balanced salt solution (HBSS), D-glucose, cell proliferation reagent WST-1, Tris/HCl pH 7.4, sodium rthovanadate (Na_3_VO_4_), phenylmethanesulfonyl fluoride (PMSF), L-glutamine, aprotinin, pepstatin, the bicinchoninic acid (BCA) kit, and puromycin (MABE343 anti-puromycin, clone 12D10 mouse monoclonal) were purchased from Sigma-Aldrich (Dublin, Ireland). Acetic acid and sodium hydroxide (NaOH) were procured from Fisher Scientific (Dublin, Ireland). Hydrochloric acid (HCl) was from VWR (Dublin, Ireland). Trinitrobenzene sulfonic acid (TNBS) reagent was from Medical Supply Co Ltd. (Dublin, Ireland). The C2C12 (subclone C2/4) mouse adherent myoblast cell line was purchased from ATCC^®^ CRL1772, Manassas, VA (Lot number 60339292), USA. Serum-free and DMEM amino acid medium was from US Biological (Salem, MA, USA). Precision Plus ProteinTM Dual Color Standards (SM1811) and sodium pyruvate (GE Healthcare, Thermo-Fisher) were both purchased from Thermo Fisher (Dublin, Ireland). Human insulin growth factor-1 (IGF-1) (100-11) was from PEPROTECH (London, UK). SDS-PAGE precast gels (4–15%) were obtained from Accuscience Ltd (Dublin, Ireland). All primary antibodies for phosphor (p)mTOR, mTOR, p4-EBP1, 4-EBP1S6 ribosomal protein, pS6 ribosomal protein, and β-actin were from Cell Signaling (Bioke, Leiden, The Netherlands). Secondary antibodies, namely green rabbit (926-32211 IRDye 800 CV) and goat anti-rabbit IgG, were obtained from LI-COR Biosciences UK Ltd (Cambridge, UK).

### Direct enzymatic hydrolysis of the sprat protein hydrolysate

BioMarine Ingredients Ireland Ltd (Lough Egish Food Park, Castleblayney, Co. Monaghan, Ireland) generated the SPH under proprietary conditions in a mode analogous to that previously reported (21).

### Simulated gastrointestinal digestion

A simulated gastrointestinal digest of SPH was generated using a modification of the method reported by Walsh et al. (22). A 2.0% (w/v) solution (on a protein equivalent basis) of SPH was changed to pH 2.0 (6 M HCL) and incubated at 37°C with pepsin at an E:S of 2.5% (w/w). Following 90 min incubation, the pH was adjusted to pH 7.0 (1 M NaOH), and pancreatin at an E:S of 1% (w/w) was added and incubated at 37°C for a further 150 min. The sample was then heated at 85°C for 15 min, then left to cool, freeze-dried, and stored at −20°C.

### Protein and AA quantification

The protein equivalent content of the hydrolysate and GI digest was determined using the macro-Kjeldahl procedure (23) using a nitrogen-to-protein conversion factor of 5.892 (21). The total and free AA content of the samples was determined using an accredited external company. The AA score was calculated as the mg of the limiting essential AA(s) per g of sample protein divided by the mg of the same AA per g of the reference protein (21).

### Physicochemical characterization

The DH of the samples was calculated by the TNBS method (21). Gel permeation high-performance liquid chromatography (GP-HPLC) and reversed-phase ultra-performance liquid chromatography (RP-UPLC) were used to determine the molecular mass distribution and peptide profiles of the SPH and its SGID-treated equivalent (24, 25).

### Color measurement

The color [L^*^ (lightness), a^*^ (redness), and b^*^ (yellowness)] values of the SPH powder were measured using a Konica Minolta CR-400 Chroma Meter (Minolta Camera Co., Osaka, Japan) as described by Cermeno et al. (26).

### Protein solubility index

The solubility of the SPH was determined at pH 2.0, 4.0, 6.0, 7.0, 8.0, 9.0, 10.0, and 12.0 in accordance with the method reported by Connolly et al. (27) with minor modifications. A 4.0% (w/v) solution (on a protein equivalent basis) of SPH was prepared in distilled water (dH_2_O, 5 mL) and stirred gently at ambient temperature (Stuart-heat stir, SB162, Keison Products Chelmsford, England) for 20 min. Aliquots of this protein suspension (9 mL in each tube) were then adjusted to pH values between pH 2.0 and 12.0 using NaOH or HCl, as required, before the total volume was adjusted to 20 mL with dH_2_O. This led to a final protein/protein equivalent concentration of 1.8% (w/v). The pH was readjusted, if necessary, following a 30 min equilibration under constant stirring. The solutions were centrifuged at 21,150 *g* for 20 min (Heittich Zentrifugen Universal 320R centrifuge, Andreas Heittich GmbH and Co., Tuttlingen, Germany). The supernatant was transferred into fresh tubes, an aliquot of the supernatant was diluted 1:100, and its protein content was estimated using the BCA assay. Protein solubility (%) was expressed as a percentage of the protein in the supernatant divided by the protein content in the starting suspension.

### Heat coagulation time

The heat coagulation time (HCT) was measured at 140°C for the SPH sample at pH 6.0 and 8.0 as described by Connolly et al. (27). An aqueous suspension of the SPH was prepared to a final concentration of 6.0% w/v (on a dry weight basis) and stirred for 1 h at ambient temperature. The pH was then adjusted and left to equilibrate for 1 h at room temperature. Following this, aliquots (2 mL, in triplicate) were added to glass tubes (10 mm i.d. 120 mm, AGB Scientific, Dublin, Ireland), sealed with silicone bungs, and submerged in an oil bath set at 140°C (Elbanton BV, Kerkdriel, The Netherlands). HCT was recorded as the time (in sec) required for the visible onset of coagulation of the sample to occur. All heat stability tests were performed in triplicate.

### Emulsification properties

The emulsifying capacity of the hydrolysates was established at pH 2.0, 4.0, 6.0, 8.0, 10.0, and 12.0 as per Connolly et al. (27) with minor modifications. A final concentration of 0.05 g 100 mL^−1^ hydrolysate solution (on a dry weight basis) was prepared in distilled water after pH adjustment with NaOH or HCl. A 28 g sample of the hydrolysate dispersion and 12 g of sunflower oil (obtained from a local food store) with Sudan III (40 mg L^−1^ of oil) were placed in a 50 mL centrifuge tube, and homogenization was carried out using an Ultra-Turrax^®^ T25 homogenizer (IKA^®^ Werke GmbH and Co. KG, Staufen, Germany) at 16,000 rpm for 60 s. An 0.100 mL aliquot of the emulsion formed was immediately diluted 1: 800 with 0.1% (w/v) sodium dodecyl sulfate (SDS), and the absorbance was established at 500 nm using a UVmini-1240 spectrophotometer (Shimazu, Canby, USA), against a 0.1% (w/v) SDS blank for determination of the emulsion activity index (EAI). The remaining emulsion was stored upright and undisturbed in the 50 mL tube at room temperature. After 30 min, an aliquot (0.1 mL) from the lower portion of the tube was taken and diluted 1:800 with 0.1% (w/v) SDS to quantify the emulsion stability (ES).

The EAI was estimated as follows:


$$\begin{array}{l} \text{EAI }\left({\text{m}}^{2}{\text{g}}^{-1}\right)=2\times 2.303\text{Ab}{\text{s}}_{500}\times \text{Dilution Factor}/\text{l}\left(1-\text{Φ}\right)\text{c }10,000 \end{array}$$

Where, Abs_500_ is the absorbance at 500 nm, l the light path length (cm), Φ the oil volume fraction, and c the hydrolysate concentration (g 100 mL^−1^).

The ES after 30 min holding was expressed as follows:

% ES = (Abs_500_ bottom half of emulsion stored for 30 min/Abs_500_ of freshly prepared emulsion) × 100

All emulsion analyses were carried out in triplicate at room temperature.

### *In vitro* antioxidant analyses

#### ORAC assay

The ORAC assay was carried out using the method described previously (28). A volume of 50 μL of the blank (0.075 M sodium phosphate buffer pH 7.0), standards (10–120 μM Trolox), and test samples at a final concentration of 0.2 mg protein equivalent.mL^−1^ was mixed with 0.78 μM fluorescein (50 μL) and incubated at 37°C for 15 min. The reaction was initiated by addition of 25 μL of 0.221 M AAPH, and the fluorescence (Ex/Em wavelengths of 485/520 nm) was measured every 5 min over a 2 h period at 37°C. The ORAC values were reported as μmol of Trolox equivalents per g sample (μmol TE/g sample).

#### FRAP assay

The FRAP activity was determined as described previously (28). A volume of 150 μL of FRAP reagent [0.3 M acetate buffer (pH 3.6), 0.01 M 2, 4, 6-tripyridyl-s-triazine (TPTZ), and 0.02 M FeCl3.6H_2_O] was added to a microplate, and the absorbance (590 nm) was determined using a plate reader (BioTek Synergy HT, Waltham, MA, USA). An aliquot (20 μL) of 0.3 M acetate buffer pH 3.6 (blank), standards (0–200 μM Trolox), and test samples at final concentrations of 10 mg protein.mL^−1^ were added, and the absorbance was measured after incubation at 37°C for 30 min. The FRAP values were reported as μmol of TE/g sample.

### Cell culture

Undifferentiated C2C12 myoblasts were kept in a growth medium (DMEM), which included 10% (v/v) fetal bovine serum (FBS), 1% (v/v) penicillin/streptomycin, and 1% L-glutamine, in a humidified atmosphere containing 5% CO_2_ at 37°C. As previously mentioned, differentiation was induced by placing 80% confluent cell cultures in a differentiation medium, which is DMEM-supplemented with 2% heat-inactivated horse serum (29). Before the main treatment, fully differentiated myotubes were starved of nutrients for 1 h in DMEM AA and serum-free medium containing 1 mM sodium pyruvate, 1% (v/v) penicillin/streptomycin, 1 mM L-glutamine, 6 mM D-glucose, and 34 mM NaCl (pH adjusted to 7.3). The C2C12 cells were then treated with the SGID-SPH in DMEM AA and serum-free medium at a final concentration of 1 mg protein equivalent/mL for 4 h. As a negative control for the assay, cells were incubated with DMEM AA and serum-free medium alone, while 100 ng/mL human insulin growth factor-1 (IGF-1) was used as a positive control.

#### WST-1 cell viability assay

Cells were seeded and differentiated in clear 96-well plates at a concentration of 7 × 10^3^ cells/well in 100 μL. On full differentiation, the cells were washed and changed to DMEM AA and serum-free medium for 1 h. Each well was then supplemented with DMEM AA, serum-free media (as a negative control), or medium conditioned with various concentrations (0.1, 0.5, and 1 mg protein equivalent.mL-1) of SGID-treated hydrolysate. The plates were then incubated for 4 h at 37°C in a humidified atmosphere containing 5% CO_2_. Following this, the WST-1 cell viability assay was conducted by adding the reagent as per the supplier's instructions (Sigma-Aldrich). Viable cells were evaluated by measuring absorbance at 450 nm. Cell viability was normalized with the number of control cells (DMEM AA and serum-free medium) and presented as percentage viability. This experiment was carried out in triplicate.

### Cellular antioxidant assay

#### Cell viability in the presence of two oxidative stress inducers (AAPH and H_2_O_2_)

Cells were seeded and differentiated in black 96-well clear bottom plates at a concentration of 7 × 10^3^ cells/well in 100 μL. Fully differentiated cells were washed with HBSS. Subsequently, HBSS medium alone (negative oxidation control) and different concentrations of AAPH (0–1,000 μM) and H_2_O_2_ (0^−^400 μM) were added to each well and incubated for 4 h. The WST-1 cell viability assay was carried out following the suppliers' instructions. Viable cells were evaluated using absorbance measurements at 450 nm. Cell viability was expressed as a percentage of viable cells in the negative oxidation control (HBSS medium alone). The experiment was performed in triplicate.

### Intracellular ROS assay

The intracellular formation of ROS was determined using DCFH-DA as described by Yarnpakdee et al. (30) with some modifications. In brief, C2C12 cells were seeded in 96-well plates at a concentration of 7 × 10^3^ cells/well in 100 μL. DCFH-DA was initially prepared in dimethyl sulfoxide (DMSO) at a concentration of 4 mM and then further diluted to 100 μM in HBSS immediately prior to its application. The C2C12 myotube cells that were fully differentiated were rinsed with HBSS (100 μL/well, three times), followed by treatment with 1 mg protein equivalent.mL^−1^ of SPH-SGID (100 μL/well) for 1 h. A positive control, consisting of Trolox at a final concentration of 100 μM, was conducted under the same conditions. A 100 μL aliquot of medium containing the test substances and controls was taken out and replaced with 100 μL of media containing 75 μM H_2_O_2_ and 100 μM DCFH-DA in HBSS. The fluorescence of the resulting 2′,7′-dichlorofluorescein (DCF) product was then measured every 10 min at 37°C for 90 min using a plate reader (BioTek Synergy HT, Waltham, MA, USA) with excitation at 485 nm and emission at 535 nm. Oxidation control wells consisted of cells in the presence of DCFH-DA and H_2_O_2_ without hydrolysate. The intracellular ROS level obtained in the presence of the hydrolysate was expressed as a percentage of the relative fluorescence intensity of the oxidation control cells.

### Electrical impedance measurement

Label-free, non-invasive, electric impedance measurements were taken using an xCELLigenceTM RTCA Instrument (ACEA Biosciences, Inc., San Diego, CA, USA) using a microelectronic E-16 well gold plated base sensor plate (ACEA Biosciences) (31). C2C12 myoblasts were cultured on an electrode-containing plate, as previously described (32). Throughout the cycle from myoblast proliferation through myotube formation, an automated reading of cell status, indicated as cell index (CI), was taken in real time (every 15 min during myoblast proliferation and every 2 min during myotube formation). Data were normalized to the start of the treatment phase of the experiment.

### Myotube diameter measurement

After 4 h incubation with controls and the SPH-SGID, multinucleated myotubes were counted under a phase-contrast microscope (Olympus CKX31, Tokyo, Japan). Image J software (National Institutes of Health, Baltimore, MD) was used to analyze images to quantify changes in myotube thickness. For each treatment condition, three diameter measurements were collected along each myotube, totaling at least 100 myotubes across at least six different fields. The average myotube diameter (μm) was then used to represent each treatment condition in the analysis.

### Western blot analysis

Cells were seeded and differentiated in 6-well plates in a final volume of 2 mL cell culture medium/well. Trypsinization was used to harvest cells. These cells were washed three times with PBS (200 μL/well) and then lysed with cold lysis buffer (10 mM Tris/HCl pH 7.4, 150 mM NaCl, NaF, and 1% Na_4_P_2_O_7_) containing phosphatase inhibitor [Na_3_VO_4_ (1 mM) and protease inhibitors (phenylmethanesulfonylfluoride fluoride (PMSF) (1 mM), pepstatin (1 μM), and aprotinin (1.5 μg.mL^−1^)] for 30 min on ice, and then, each plate was scraped using a cell scraper. The homogenates obtained were then centrifuged at 130 *g* at 4°C for 10 min to remove nuclei and cellular debris. After determination of the protein content using the Bradford assay (following the suppliers' instructions), the lysates (30 μg protein/lane) were loaded on stain-free 4–15% linear gradient SDS polyacrylamide gel electrophoresis (PAGE) precast gels, and the gel was transferred to a nitrocellulose membrane using the semi-dry transfer technique (Trans-blotR TurboTM; Bio-Rad). After blocking with 5% (w/v) skimmed milk powder in 1X Tris-buffered saline (TBS) containing 0.5% Tween-20 (TBST) for 1 h at 37°C, the membrane was incubated with each antibody (1:1000 in 5% BSA in TBST: mTOR, phosphor (p)mTOR, P70S6K, pP70S6K, 4-EBP1, p4-EBP1, S6 ribosomal protein, pS6 ribosomal protein, β-actin, and puromycin) at 4°C overnight. The membrane was first treated with primary antibodies, except for puromycin which received a goat anti-mouse IgG2a-specific antibody, and then incubated with a secondary green rabbit antibody for 1 h. Images were captured using a UVITEC Cambridge Imaging system, and whole-lane band densitometry was quantified using NineAlliance UVITEC Software. After probing with phosphorylated antibodies, the membranes were stripped according to the manufacturer's instructions and then re-probed with total antibodies. Phosphorylated proteins were normalized to their respective total protein, while puromycin was normalized to the total protein density obtained from the stain-free lane for quantification purposes.

### Muscle protein synthesis

MPS was determined *via* the surface sensing of translation technique (SUnSET) (33). Following 1 h nutrient deprivation in AA and serum-free DMEM medium, differentiated C2C12 myotubes were treated with either 1 mg protein equivalent.mL^−1^ SPH in AA and serum-free DMEM medium containing 1 μM puromycin (Merck Millipore Limited) and 100 ng.mL^−1^ IGF-1 (positive control) or in amino acid and serum-free DMEM (negative control) containing 1 μM puromycin for a further 4 h. Immunoblotting was then used to assess MPS after obtaining the cellular protein lysates.

### Statistical analysis

The results are presented as the mean ± standard deviation (SD) of three independent experiments. Data were analyzed using one-way or two-way ANOVA followed by Tukey's *post hoc* analysis. The SPSS software program (Version 27, IBM Inc., Chicago, IL, USA) was used to perform statistical analyses on the data. In all analyses, a *P* < 0.05 was taken to indicate statistical significance.

## Results and discussion

### Properties and physicochemical characteristics of the SPH

The protein content of the off-white SPH powder sample was 84.71 g/100 g. Following the terminology used for milk proteins, this product would correspond with an SPH80 since it contains more than 80% (w/w) protein. The DH of the SPH was 39.71 ± 0.24%, and this did not significantly change following simulated *in vitro* digestion of the sample (SPH-SGID) (Table 1). The molecular mass distribution of the SPH and SPH-SGID samples showed a high content of low molecular mass peptides (<1 kDa), followed by the fraction with a molecular mass between 1 and 5 kDa with very low levels for peptides >10 kDa (Table 1). The peptide profiles were similar in the SPH and SPH-SGID samples (data not shown) following reversed-phase ultra-performance liquid chromatography (RP-UPLC) analysis.

**Table 1:** The degree of hydrolysis (DH) and molecular mass distribution profiles of the sprat (*Sprattus sprattus*) protein hydrolysate (SPH) pre- and post-simulated gastrointestinal digestion (SPH-SGID).

**Test sample**	**DH (%)**	**>10 kDa**	**5-10 kDa**	**1-5 kDa**	** < 1 kDa**
**Molecular mass distribution (%)**					
SPH	39.71 ± 0.24	0.58	1.94	8.78	88.7
SPH-SGID	41.05 ± 0.24	0	0.1	9.7	91.2

Data reported as mean ± SD (n = 3).

Table 2 provides the AA composition of the SPH sample and shows that the hydrolysate contains all the essential and non-essential AAs. The most abundant AAs were Glx (137 g/kg^−1^) and Asx (91.5 g/kg^−1^), and the lowest level was found for Cys (5.9 g/kg^−1^). The protein quality of a dietary protein source can be estimated by comparing it to three reference AA scores of a model protein in accordance with the requirements of different age groups (34). These include the AA requirements for (1) infants < 6 months old (2) children 6–36 months old, and (3) children >36 months, adolescents, and adults. In this study, the ratios of each of the essential AA in the SPH with respect to the levels highlighted for each of the three reference cohorts were determined, and the lowest ratio was associated with the AA score. The manner in which the essential AA levels in a protein compare to those of a reference protein as well as which AA(s) may be limiting in the protein can be determined by AA score evaluation.

**Table 2:** Amino acid (AA) profile of the sprat (*Sprattus sprattus*) protein hydrolysate (SPH).

	**AA**	**AA Content of SPH**
**Non-essential (NEAA**	**Cys**	5.9
	**Arg**	**58.8**
	Asx	91.5
	Pro	35
	Ser	38.2
	Glx	137
	Gly	48
	Ala	55.6
	Tyr	28.3
**Essential amino acids**	Val	37.9
**(EAA)**	Ile	28.2
	Leu	59.4
	Trp	7
	Phe	28.6
	His	30
	Lys	82.8
	Met	22.2
	Thr	39.8
	EAA	335.9
	NEAA	498.3
	EAA:NEAA	0.7
	BCAA	125.5
	TAA	834.2

AA residues denoted by their 3-letter code. Asx, aspartic acid and asparagine; Glx, glutamic acid and glutamine; EAAs, essential AAs; NEAA, non-essential AAs; BCAA, branched chain AAs; TAA, total AAs.

Table 3 shows the calculated AA scores for the SPH in different population cohorts. Tryptophan was identified as the limiting essential AA (0.49) when compared against the recommended AA requirement pattern for infants <6 months (34). Tryptophan was also identified as the limiting essential AA with an AA score of 0.97 for the cohort of children from 6 months to 3 years old.

**Table 3:** Calculated amino acid (AA) scores for the sprat (*Sprattus sprattus*) protein hydrolysate (SPH) for different population cohorts.

**Populations**		**Amino**	**acid ratio**						
	**SAA**	**Trp**	**Thr**	**Val**	**Ile**	**Leu**	**AAA**	**Lys**	**His**
Infant (up to 6 months)	1.07	0.49^*^	1.07	0.81	0.61	0.73	0.71	1.42	1.69
Child (6 months to 3 years)	1.23	0.97^*^	1.52	1.04	1.04	1.06	1.29	1.71	1.77
Older child, adolescent, adult	1.44	1.25	1.88	1.12	1.11	1.15	1.64	2.04	2.21

^*^AA score (1st limiting amino acid), AA residues are denoted by their three-letter code. SAA, sulfur AAs; AAA, aromatic AAs.

Therefore, this information indicates that SPH may need to be supplemented, at different levels, with AA in order to meet the AA requirements of these two population cohorts. All AA scores for the older children, adolescent, and adult population cohort were above a value of 1.0 which indicates that the level of all essential AAs in the SPH met those recommended for this cohort. However, protein quality scores (which are measured in relation to the digestibility and the bioavailability of the essential AAs in a given protein) would need to be evaluated in order to confirm that SPH definitively meets the human dietary requirements for the specific population cohorts.

### Technofunctional properties of SPH

#### Color measurement

Color is a critical criterion in the consumer perception of food formulations and is one of the many challenges associated with the utilization of fish as sources of functional ingredients. Ideally, fish protein ingredients produced at an industrial scale should be color- and odor-free, and studies have been reported on optimizing these requirements *via* enzymatic hydrolysis during the extraction of protein (35). The SPH was pale yellow in color with L^*^, a^*^, and b^*^ values of 77.80, 6.17, and 31.51, respectively. The final color of FPHs depends on multiple factors such as raw material composition, the enzymes used, and the parameters used for hydrolysis as reported by Egerton et al. (36). Blue whiting protein hydrolysates (BWPHs) were previously reported to have L^*^ values in the range 70 to 72 (37) which were lower than that of the SPH herein. Moreover, the a^*^ and b^*^ values for the BWPHs were in the range of 0.28 to 0.49 and 7.95 to 9.90, respectively. Jemil et al. (38) assessed the color properties of four different FPHs (sardinella, zebra blenny, goby, and ray). The L^*^ values of samples were in the range 65.30 to 80.02, b^*^ values were between 26.81 and 47.39, and a^*^ were between −6.18 and 6.49. The dark color of the FPHs could be attributed to the oxidation of myoglobin and melanin in the raw materials (39). The color of the SPH is imperative for its final applications. For instance, using an SPH ingredient that undergoes color development on heating at high temperatures may affect the final color of the processed products. Compared to other reported fish sources, the sprat hydrolysate was relatively lighter in color and therefore can be widely applied as an ingredient in multiple formulations without the need for further processing.

### Protein solubility

**Figure 1:**
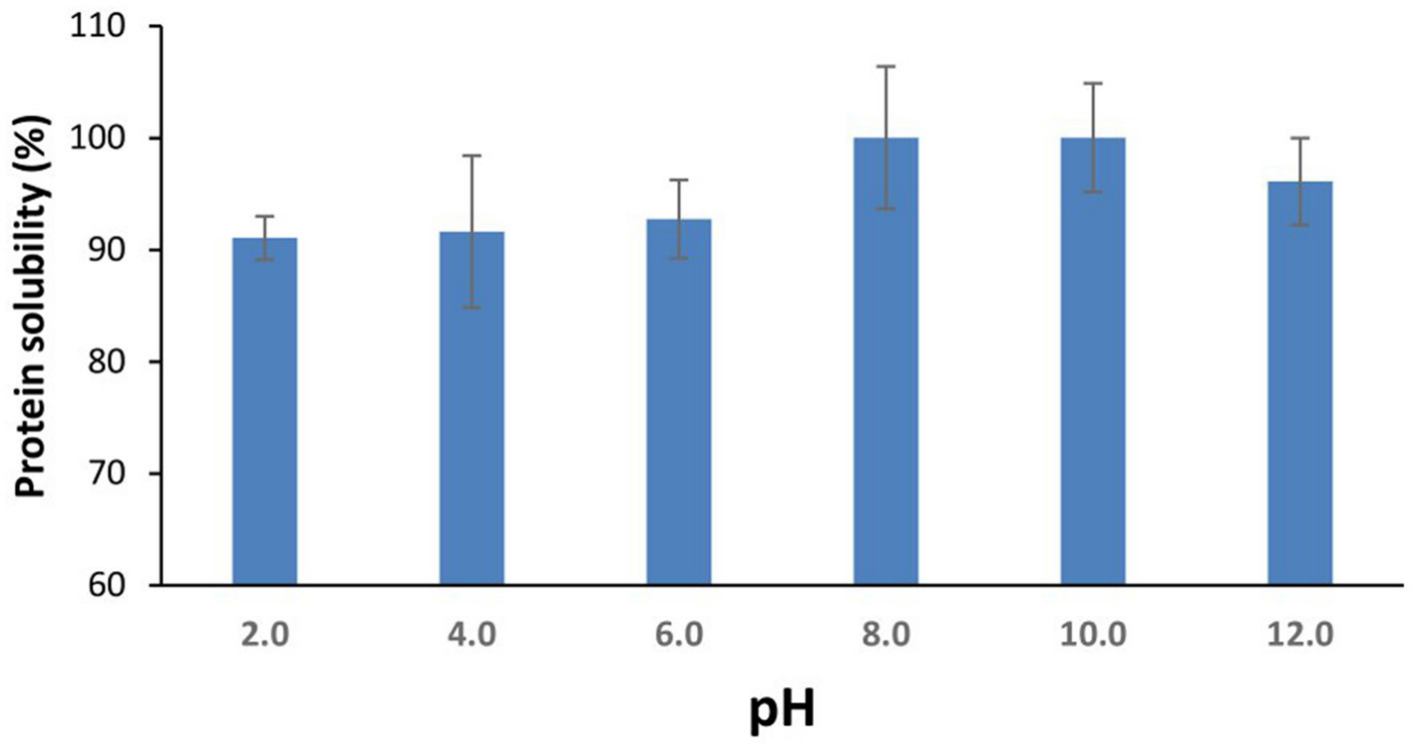
Aqueous protein solubility (%) of the sprat (*Sprattus sprattus*) protein hydrolysate as a function of pH. Values represent mean ± SD (*n* = 3).

The solubility of proteins and their hydrolysates is an important technofunctional requirement for their applications in the food and pharmaceutical industries. Protein structure, pH, ionic strength (and salt type), and temperature all affect protein solubility (40). The aqueous solubility of the SPH was >90% across the pH range (pH 2.0–12.0) tested herein. While the mean solubility value at pH 8.0 was the highest, there were no statistically significant differences (*p* > 0.05) in solubility between the different pH values (Figure 1). A similar solubility profile was reported for protein hydrolysates from blue whiting (BWSPH), which had > 80% solubility between pH 2.0 and 10.0 (36). The overall high solubility is consistent with the solubility results observed for yellow stripe trevally (*Selaroides leptolepis*) (20) and sardinella, zebra blenny, goby, and ray protein hydrolysates (38).

The high solubility of the SPH could result from the large proportion of low molecular mass peptides (<1 kDa) contained therein. Furthermore, hydrolysis increases the number of terminal polar (NH3 COO-) groups and results in the unfolding of the protein structure revealing polar AA groups (such as serine, threonine, cysteine, tyrosine, asparagine, and glutamine) which can then interact with water molecules thereby enhancing solubility (41, 42).

### Heat stability

Heat stability is an important parameter in the development of protein ingredients as it indicates changes in protein stability due to thermal treatments such as cooking. The HCT depends on several factors such as protein concentration, protein/peptide conformation, as well as the charge and structure of proteins. The impact of two different pHs on the HCT of 6% (w/v) SPH at 140°C was assessed herein. The HCT increased from 25 to 40 s by increasing the pH from pH 6.0 to 8.0. Enzymatic hydrolysis was shown previously to enhance protein/hydrolysate ingredient heat stability. The enzymatic hydrolysis of brewers' spent grain protein-enriched isolates showed a significant increase in heat stability when going from pH 6.0 to 8.0, i.e., increasing from 40 ± 3 s to >300 min (27). Ryan et al. (43) also reported the effect of pH on HCT at 140°C of soy protein hydrolysates where the HCT significantly increased with increasing pH (43).

### Emulsion properties (EAI and ES)

**Figure 2:**
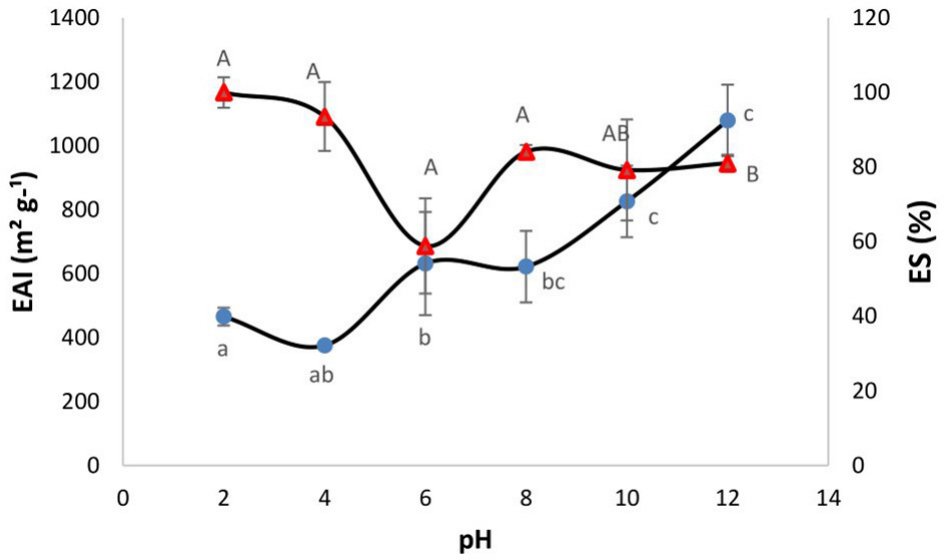
Effect of pH on the emulsion activity index (EAI -•) and emulsion stability (ES % -Δ) of the sprat (*Sprattus sprattus*) protein hydrolysate. Values represent mean ± SD (*n* = 3). Different lowercase letters show significant differences for EAI at different pHs (*p* < 0.05). Different uppercase letters show significant differences for ES at different pHs (*p* < 0.05).

The emulsion activity is a measure of the potential of an ingredient to be surface active and stabilize product formulations that contain both lipophilic and hydrophilic phases such as mayonnaise, salad dressings, sauces, milk, and sausages. Proteins and protein hydrolysates can stabilize emulsions by forming a viscoelastic film which prevents the phases from separation when absorbed to the surface of oil droplets during the emulsification process (44). Figure 2 shows the EAI and ES values for the SPH as a function of pH. Overall, the SPH showed higher EAI values at higher pH (pH 10 and 12 with EAI values of 825.59 and 1079.12 m^2^ g^−1^, respectively) and the lowest EAI was obtained at pH 4.0 (375.42 m^2^ g^−1^). Similar emulsion activity values have been reported for hydrolysates generated from marine-based protein sources, e.g., from *Nemipterus japonicus* (45). As can be observed in Figure 2, the ES of the SPH was between 58 and 100% across the pH range 2.0–12.0. The ES of the SPH was at its lowest at pH 6.0 (58.9%), and this was potentially related to the trend of a decrease in solubility at this pH. The ES increased with increasing pH which may be related to the trend of an increase in solubility (although non-significant in the present instance). Pacheco-Aguilar et al. (46) showed that the emulsion capacity (EC) of hydrolysates formed from Pacific whiting (*Merluccius productus*) muscle was significantly influenced by DH and pH, except at pH 4. Higher ES and EAI values were reported at higher pH values (46). According to Villamil et al. (47), the partial hydrolysis of proteins changes their structure and enhances the flexibility of the resulting peptides that subsequently align to the interface to form and stabilize emulsions. However, it is well recognized that extensive hydrolysis may reduce emulsifying properties (48). In addition, in an alkaline medium rich in negative charge, polypeptides unfold and expose hydrophobic groups facilitating protein/lipid interactions and improving the diffusion and stabilization at the oil/water interface (49).

Overall, the technofunctional data presented herein showed that the SPH had good solubility across a broad range of pH values. It had a light yellow color. It had an HCT of 40 s at pH 8.0. Moreover, compared to other studies, the SPH herein showed much higher emulsion activity and stability. The SPH did not display any foaming ability. These results indicate that the SPH has properties providing it with significant versatility for different product applications.

### *In vitro* antioxidant activity

The ORAC and FRAP activities of the SPH and its simulated GI digests are presented in Table 4. Prior to SGID, an ORAC value of 587.49 ± 15.23 μmol TE/g sample was obtained, and a similar ORAC activity was observed following SGID (Table 4). As previously stated, the DH data and molecular mass distribution results indicate that limited hydrolysis occurred during SGID, which indicates that treatment with the gastrointestinal enzymes did not release more peptides with higher ORAC activity from the precursor peptides. However, it is possible that hydrolysis of peptides during SGID may have resulted in both a loss and gain of specific bioactivities, resulting in no overall change in the total bioactivity of the peptides in the SGID-treated SPH. The ORAC antioxidant activity of the SPH was higher than the ORAC activity described for other fish protein hydrolysates from channel catfish, pacific hake, blue mussel (*Mytilus edulis*), and blue whiting sources with ORAC values of 16, 225, 66.26, and 121.56 μmol TE/g sample, respectively (50–52). The SPH ORAC values observed herein were lower than the range described for Atlantic salmon (*Salmo salar*) trimming protein hydrolysates (601.47–882.58 μmol TE/g sample) (53).

**Table 4:** *In vitro* oxygen radical absorbance capacity (ORAC) and ferric reducing antioxidant power (FRAP) activity, and *in situ* C2C12 cellular reactive oxygen species (ROS) production of sprat (*Sprattus sprattus*) protein hydrolysate (SPH) pre- and post-simulated gastrointestinal digestion (SPH-SGID).

**Sample**	**ORAC value (μmol TE/g sample)**	**FRAP value (μmol TE/g sample)**	**ROS production (%, H_2_O_2_)**
SPH	587.49 ± 15.23	10.93 ± 0.46^*^	nd
SPH-SGID	549.42 ± 8.48	5.29 ± 0.15	67.64 ± 2.32

Data reported as mean ± SD (n = 3). The ROS value in the presence of H_2_O_2_ was expressed as a percentage relative to the control. ^*^Indicates a significant difference at a p-value of < 0.05 between pre- and post-simulated gastrointestinal digestion values. nd, not determined. TE, Trolox equivalent.

FRAP values ranging from 10.93 ± 0.46 to 5.29 ± 0.15 μmol TE/g sample were obtained for the SPH prior to SGID and SPH-SGID, respectively (Table 4). The FRAP activity of the SPH was significantly decreased following SGID (Table 4). Similar findings were reported for other FPHs, bovine protein hydrolysates, and whey protein hydrolysates which had reduced FRAP level post-SGID compared to pre-SGID (50, 54, 55).

The differences observed in the antioxidant activity using the FRAP and ORAC assays may be explained due to the different modes of activity used in the analysis of these assays. The ORAC assay measures the capacity of test compounds to scavenge peroxyl radicals through hydrogen atom transfer (HAT), whereas the FRAP assay is categorized as an electron transfer (ET)-based, non-radical assay technique (56).

### Cell line experiments

#### Cell viability assay (WST-1)

**Figure 3:**
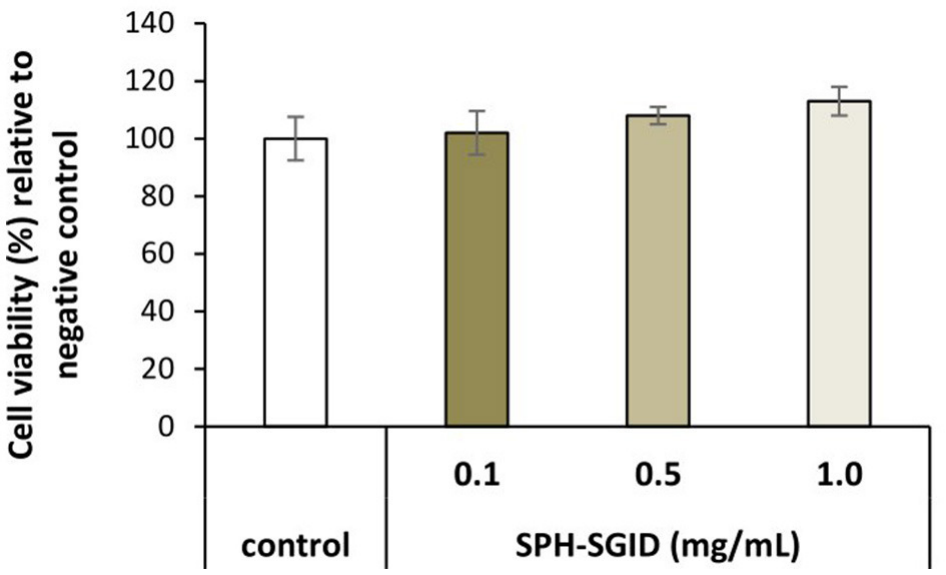
Viability of muscle myotube (C2C12) cells treated with 0.1, 0.5, and 1.0 mg protein equivalent.mL^−1^ sprat (*Sprattus sprattus*) protein hydrolysate subjected to simulated gastrointestinal digestion (SPH-SGID). The negative control was amino acid and serum-free media without SPH. Treatment of cells was for 4 h after 1 h of nutrient deprivation. The results represent mean ± SD (*n* = 3) and are expressed relative to the negative control.

Differentiated myotubes were nutrient deprived for 1 h and then incubated with AA and serum-free media as a control and treatment with 0.1, 0.5, and 1 mg protein equivalent.mL^−1^ of SPH-SGID for 4 h. After 4 h of incubation with various concentrations of SPH-SGID, the cell viability, as determined by the WST-1 assay, ranged from 102 to 113% compared to the control conditions (Figure 3). Treatment of cells with up to 1 mg protein equivalent.mL^−1^ SPH-SGID for 4 h after 1 h of nutrient deprivation led to no negative effect on cell viability (at all time points) compared with control conditions. As there was no reduction in cell viability, an SPH concentration of 1 mg protein equivalent.mL^−1^ was used for subsequent treatments (Figure 3).

### Cellular antioxidant activity

To further evaluate the antioxidant potential of SPH, the ability of the SPH-SGID to affect the endogenous antioxidant defense systems was assessed. The results from biochemical antioxidant assays, while useful, are *in vitro* tests and therefore may not be easily extrapolated to more complicated systems such as in the human body (57). *In situ* cellular-based assays, which may more accurately represent the target site of oxidative stress *in vivo*, may therefore be more relevant in the assessment of test compound antioxidant properties. The antioxidant activity of SPH-SGID was assessed *in situ* in C2C12 differentiated myotubes on the basis of the extent of ROS generation (Table 4) in the presence and absence of a pro-oxidant. The results showed that incubation with 1 mg protein equivalent.mL^−1^ SPH-SGID for 1 h did protect C2C12 myotubes from the pro-oxidant effects of H_2_O_2_.

The application of cellular antioxidant-based assays of proteins and their derivatives using C2C12 myoblast and myotubes was reported in a number of previous studies (58–60). The cell cytotoxicity of two oxidative stress inducers (AAPH and H_2_O_2_) was pre-evaluated herein at different concentrations to investigate their potential toxic effects on C2C12 cells. The results showed that AAPH at concentrations ranging from 0 to 1,000 μM did not decrease cell viability by more than 85% which indicates that a higher concentration of AAPH was needed to decrease cell viability and induce more oxidative stress in C2C12 myotubes. Therefore, further studies are required to optimize the concentration of AAPH required to mediate a cytotoxic effect. There are several studies which employ H_2_O_2_ for the induction of oxidative stress in C2C12 cells (61, 62). Different concentrations of H_2_O_2_ (0–400 μM) resulted in a decrease in cell viability ranging from 35 to 85%. A toxic effect yielding <75% cell viability was found at ≥ 75 μM H_2_O_2_. Therefore, H_2_O_2_ was selected to represent the oxidative stress inducer at a concentration of 75 μM giving a mean cell viability value herein of 74%.

The cellular antioxidant assay was carried out to determine the potential antioxidative properties of the SPH-SGID against H_2_O_2_-induced intracellular ROS generation, as per Yarnpakdee et al. (30). The commercial antioxidant Trolox, which was used as a positive control, significantly reduced ROS generation as compared to the negative control, H_2_O_2_-stressed cells. Intracellular ROS generation in the SPH-SGID-treated cells (1 mg protein. mL−1 equivalent) was 67%. This demonstrated that treatment with SPH-SGID led to significantly (*p* < 0.05) lower levels of ROS generation compared to the negative control (Table 4). The significant reduction in ROS generation in the C2C12 cells treated with the SPH-SGID concurred with the results observed in the *in vitro* ORAC assays (Table 4).

In accordance with the antioxidant activity observed for the SPH herein, blue whiting (*Micromesistius poutassou*) protein hydrolysates and large yellow croaker (*Pseudosciaena crocea*) protein hydrolysates (MW < 3 kDa), which contained a high content of low MW peptides, presented ${\text{O}}_{2}^{-\cdot }$ and DPPH scavenging activity *in vitro*. Furthermore, they increased the activity of the antioxidant enzymes glutathione peroxidase (GSH-Px), superoxide dismutase (SOD), and catalase (CAT) in H_2_O_2_-induced oxidative stress in HepG2 cells (50, 63). In addition, numerous fish-derived peptides have demonstrated the ability to modulate oxidative stress pathways *in vitro* (64–67). Therefore, marine proteins are potential raw materials for the generation of antioxidant peptides with the ability to enhance health by reducing oxidative stress.

No studies appear to have been previously reported on the potential antioxidant activity of FPHs in oxidative stressed muscle cells. The results obtained for SPH herein demonstrate that this sample may have potential application as an antioxidant agent. Excessive production of ROS due to redox imbalance and impaired antioxidant defense systems leads to oxidative damage in most organs, including muscle (68–70). Increased ROS buildup causes oxidative stress, which in turn encourages proteolysis and causes muscle atrophy. ROS are crucial mediators of numerous signaling pathways that control this process (71, 72).

Therefore, the application of FPHs with potential antioxidant ability to reduce oxidative stress, thereby controlling oxidative stress and regulating the redox system, may be considered a promising approach for preserving muscle function.

### Myotube growth and size

**Figure 4:**
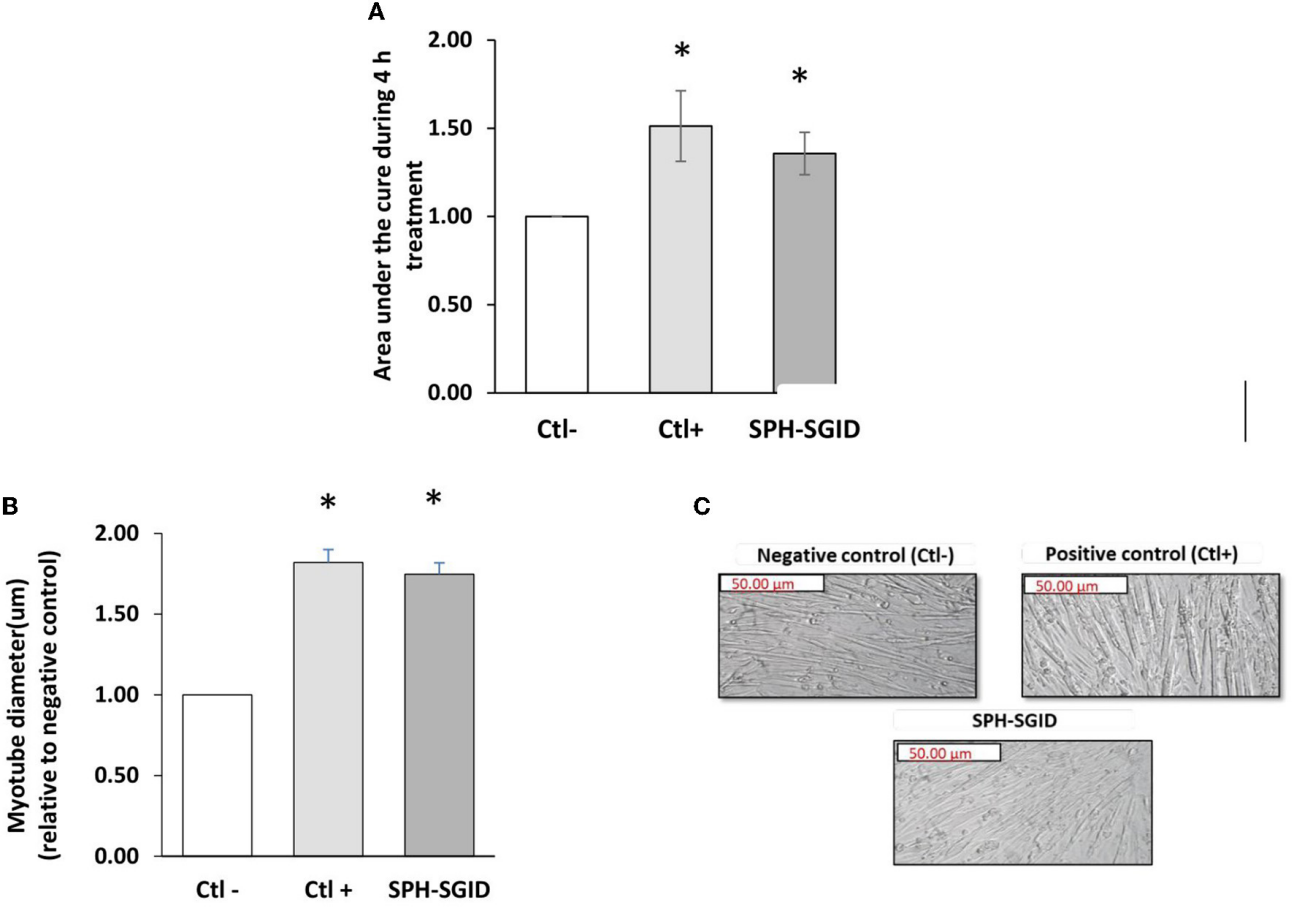
Effect of sprat (*Sprattus sprattus*) protein hydrolysate subjected to simulated gastrointestinal digestion (SPH-SGID) treatment on cell index (area under the curve) and myotube diameter in skeletal muscle cells. C2C12 myotubes were nutrient deprived for 1 h followed by 4 h treatment with 1 mg protein equivalent.mL^−1^ of SPH-SGID. Myotube growth was monitored every 2 min over 4 h. **(A)** Representative graph comparing myotube growth (cell index and area under the curve) in the presence of sample relative to the negative control. **(B)** Representative graph comparing myotube diameter in the presence of sample relative to the negative control. **(C)** Quantification of myotube diameter 4 h post-treatment as measured by microscopy. Images of myotubes treated with sample were taken at 4X magnification following 4 h treatment. All values are expressed as mean ± SD (*n* = 6). A *p*-value of < 0.05* compared to the negative control (amino acid and serum-free media). Ctl-: negative control (amino acid and serum-free media), Ctl+: positive control (100 ng.ml^−1^ IGF-1), SPH-SGID: sprat protein hydrolysate subjected to simulated gastrointestinal digestion.

C2C12 myotubes were treated with media conditioned with 1 mg protein equivalent.mL^−1^ of SPH-SGID, AA and serum-free media, and IGF-1 to investigate the potential of SPH-SGID to stimulate myotube growth and activate MPS, as previously described (29, 32, 73). Based on the data from xCELLigence^TM^ analysis, the area under the curve (AUC) of cell index (CI) was significantly increased in response to treatment with SPH-SGID compared to the negative control (*p* < 0.05) as was the case for the IGF-1 positive control treatment (Figure 4A).

Myotube thickness was assessed after a 4 h treatment to verify changes in myotube diameter based on CI results. Myotube diameter was significantly increased (*p* < 0.001) compared to the negative control with SPH-SGID treatment (Figure 4B). These results indicate that SPH may have the potential to enhance myotube growth which may lead to the stimulation of MPS. These results are similar to the findings of Lees et al. (16) which previously showed that C2C12 cells demonstrate greater hypertrophy with BWPH-fed serum taken from healthy older adults compared with non-essential AA-fed serum based on myotube thickness (*p* = 0.028). Moreover, the current results demonstrate the potential ability and comparability of SPH with BWPHs regarding the promotion of myotube growth, proliferation, and MPS and demonstrate the potential of FPHs as a functional ingredient for the improvement of muscle health (32).

### Muscle protein synthesis

The mammalian target of rapamycin (mTOR) pathway is one of the most well-known signaling mechanisms in modulating MPS. A serine/threonine kinase called mTOR detects changes in the environment and within cells, such as the availability of nutrients and the level of energy (74). mTORC1 is known as a key regulator in controlling skeletal muscle mass following contraction and mechanical load-induced hypertrophy, synergistic ablation, myotube hypertrophy, and AA sensing, in which mTOR is involved in both skeletal muscle hypertrophy and atrophy (75). This important signaling molecule has been implicated in numerous studies as a crucial mediator in the conversion of mTOR activation to MPS activity (76, 77).

The ability of the SPH-SGID treatment to stimulate mTORC1 signaling was assessed by measuring the phosphorylation of mTORC1 and its downstream signaling molecules 4-EBP1 and rpS6. Many studies have identified this critical signaling molecule as a key mediator in translating the activation of mTORC1 to the activation of MPS (76–78).

**Figure 5:**
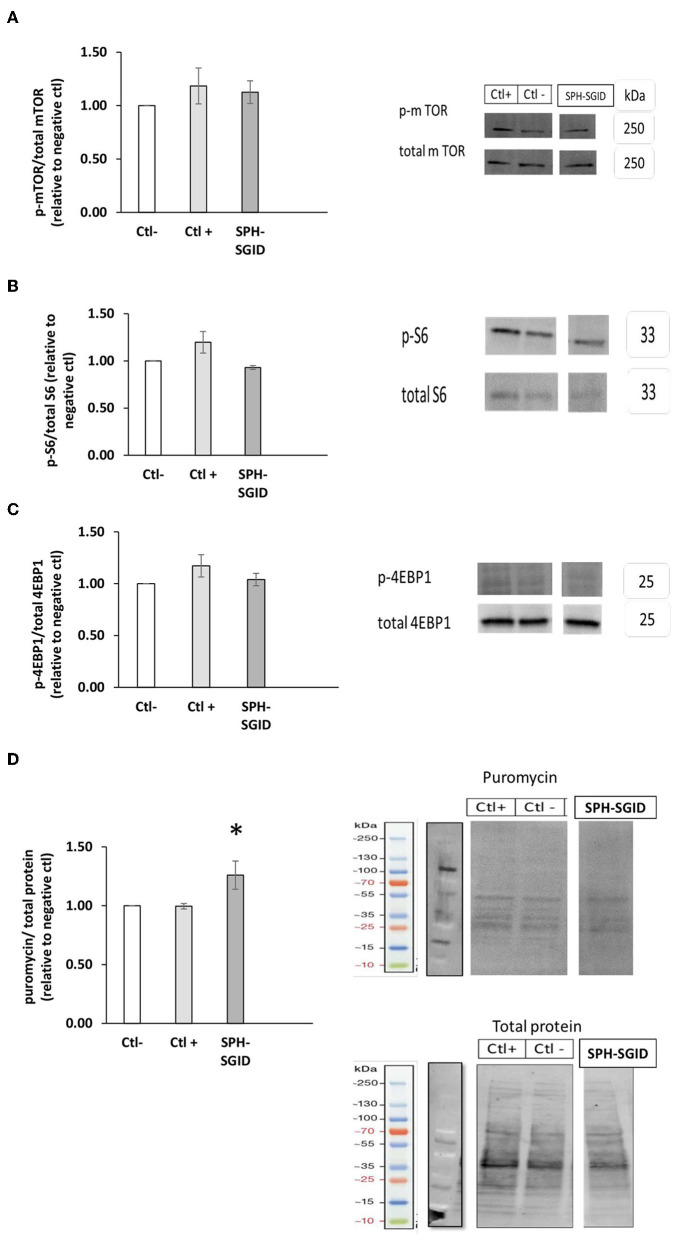
Phosphorylation of mTOR, 4E-BP1, and ribosomal S6 incubated with 1 mg protein equivalent.mL-1 sprat (*Sprattus sprattus*) protein hydrolysate (SPH) subjected to simulated gastrointestinal digestion (SPH-SGID) (*n* = 4). C2C12 myotubes were nutrient deprived for 1 h followed by treatment with SPH-SGID plus 1 uM puromycin for 4 h. Data reported as the ratio of phosphoproteins relative to the total protein. All values were expressed as a percent of the negative control within each assay. Phosphorylation of mTOR **(A)**, rpS6 **(B)**, and 4-EBP1 **(C)** following SPH-SGID treatment, and their corresponding representative immunoblot. **(D)** Muscle protein synthesis (MPS) after treatment with SPH-SGID and a representative immunoblot of MPS (measured by puromycin incorporation) relative to total protein (loading control). Data reported as mean ± SEM, *compared to negative control, *p* < 0.01. Ctl-: negative control (amino acid and serum-free media), Ctl+: positive control (100 ng. mL-1 IGF-1).

mTOR phosphorylation on treatment with SPH-SGID was not significantly different to the negative or positive controls (*p* > 0.05, Figure 5A). Furthermore, activation of the downstream targets of mTOR activation, 4E-BP1 (Figure 5B), and rpS6 (Figure 5C) was not significantly different compared to negative or positive controls (*p* > 0.05). It is possible that these markers were not sufficiently sensitive to detect changes between conditions, and a lack of upregulation of mTOR phosphorylation on C2C12 treatment with BWPHs has been previously reported (16, 32).

It has been previously reported that BWPHs stimulated MPS by activation of phosphorylated 4E-BP1 and rpS6 in C2C12 cells (32). However, while no significant changes for these markers were observed following SPH treatment, downstream markers of protein elongation and translation responded differently to SPH, suggesting that these markers—which show higher activation of protein translation, may be more sensitive to subtle anabolic differences. Lees et al. (16) observed a similar response for both mTOR and downstream markers in response to treatment with both fish and milk protein sources. However, in their study, C2C12 cells were exposed to human serum-conditioned media. This media may have other stimulators for activating the mTOR signaling pathway, such as insulin.

The SunSET technique (33) was applied herein to quantify MPS and to verify whether mTOR, rpS6, and 4E-BP1 activation could mediate an increase in MPS in skeletal muscle cells (C2C12 myotubes) following 4 h treatment with SPH-SGID. The results obtained showed that puromycin incorporation was significantly increased in response to SPH-SGID treatment compared to both the negative and positive controls (*p* < 0.05, Figure 5D). These results support the data from the CI and myotube diameter analyses. As a result, when taken as a whole, SGID boosted skeletal MPS and anabolism *in situ*, which may be due to an unknown mechanism that increased translation initiation factors rather than boosting mTOR signaling.

## Conclusion

To the best of our knowledge, this is the first report on the technofunctional properties, the *in vitro* and *in situ* antioxidant ability, and the potential effects of SPH-SGID on C2C12 myotubes. The results demonstrated the potential use of SPH as a technofunctional ingredient for different food applications due to its light color, high protein solubility, and good emulsion activity and stability when compared to results reported for other FPHs. The antioxidant activity based on the ORAC and ROS assays and the improvement in muscle growth and in MPS following SPH treatment in C2C12 cells may be of value to the marine (fish processing) sector and may contribute to the increased use of this small pelagic fish for human consumption to improve muscle health. *In vivo* studies in human volunteers are warranted to validate these findings.

## Data availability statement

The raw data supporting the conclusions of this article will be made available by the authors, without undue reservation.

## Author contributions

RF and BC: conceptualization, supervision, funding acquisition, and writing—reviewing and editing. NS: performing the cell-based experiments and writing the manuscript. NS, RF, AN, and BC: data analysis, interpretation, and statistical analyses. AN: performing the technofunctional property assessments, reading, and editing of the manuscript. PH-R and MA-B: supervision of the study, reading, and editing of the manuscript. All authors contributed to the article and approved the submitted version.
